# The Use of a Technology Acceptance Model (TAM) to Predict Patients’ Usage of a Personal Health Record System: The Role of Security, Privacy, and Usability

**DOI:** 10.3390/ijerph20021347

**Published:** 2023-01-11

**Authors:** Adi Alsyouf, Abdalwali Lutfi, Nizar Alsubahi, Fahad Nasser Alhazmi, Khalid Al-Mugheed, Rami J. Anshasi, Nora Ibrahim Alharbi, Moteb Albugami

**Affiliations:** 1 Department of Managing Health Services & Hospitals, Faculty of Business Rabigh, College of Business (COB), King Abdulaziz University, Jeddah 21991, Saudi Arabia; 2 Department of Accounting, College of Business (COB), King Faisal University, Al-Ahsa 31982, Saudi Arabia; 3 Applied Science Research Center, Applied Science Private University, Amman 11931, Jordan; 4 Department of Health Services and Hospitals Administration, Faculty of Economics and Administration, King Abdulaziz University, Jeddah 21589, Saudi Arabia; 5 Department of Health Services Research, Faculty of Health, Medicine, and Life Sciences, Maastricht University Medical Center, 6229 HX Maastricht, The Netherlands; 6 Nursing College, Riyadh Elm University, Riyadh 13244, Saudi Arabia; 7 Prosthodontics Department, Faculty of Dentistry, Jordan University of Science and Technology, Irbid 22110, Jordan; 8 Department of Business Administration, College of Business Administration (CBA), University of Business and Technology (UBT), Jeddah 23435, Saudi Arabia; 9 Department of Management Information Systems, College of Business (COB) Rabigh, King Abdulaziz University, P.O. Box 344, Jeddah 21991, Saudi Arabia

**Keywords:** eHealth, usability, adoption, privacy, security, perceived usefulness, technology acceptance model, perceived ease of use, technology acceptance, eHealth application, Saudi Arabia

## Abstract

Personal health records (PHR) systems are designed to ensure that individuals have access and control over their health information and to support them in being active participants rather than passive ones in their healthcare process. Yet, PHR systems have not yet been widely adopted or used by consumers despite their benefits. For these advantages to be realized, adoption of the system is necessary. In this study, we examined how self-determination of health management influences individuals’ intention to implement a PHR system, i.e., their ability to actively manage their health. Using an extended technology acceptance model (TAM), the researchers developed and empirically tested a model explaining public adoption of PHRs. In total, 389 Saudi Arabian respondents were surveyed in a quantitative cross-sectional design. The hypotheses were analysed using structural equation modelling–partial least squares (SEM-PLS4). Results indicate that PHR system usage was influenced by three major factors: perceived ease of use (PEOU), perceived usefulness (PU), and security towards intention to use. PHR PEOU and PHR intention to use were also found to be moderated by privacy, whereas usability positively moderated PHR PEOU and PHR intention to use and negatively moderated PHR PU and PHR intention to use. For the first time, this study examined the use of personal health records in Saudi Arabia, including the extension of the TAM model as well as development of a context-driven model that examines the relationship between privacy, security, usability, and the use of PHRs. Furthermore, this study fills a gap in the literature regarding the moderating effects of privacy influence on PEOU and intention to use. Further, the moderating effects of usability on the relationship between PEOU, PU, and intention to use. Study findings are expected to assist government agencies, health policymakers, and health organizations around the world, including Saudi Arabia, in understanding the adoption of personal health records.

## 1. Introduction

There is an extensive range of products, goods, and services covered under health information technologies (HITs), and this holds for services such as assistive technology and sensors [1,2,3,4,5,6], cloud-based services [7], electronic health records (EHRs) [8,9,10,11,12,13,14], mobile health technologies [15,16,17,18,19,20,21,22,23], medical devices, telemonitoring tools, and telehealth [3,24,25,26,27,28,29,30]. All the technologies mentioned above bring about the gathering, sharing, and usage of health information by individuals, healthcare personnel, and community-based healthcare institutions [1,8,11].

Personal health record (PHR) is defined as “An electronic application through which individuals can access, manage and share their health information, and that of others for whom they are authorized, in a private, secure, and confidential environment” [31]. PHRs represent an attractive and developing technology within the health systems and applications that has been gaining ground among different countries [32]. More specifically, PHR systems are information systems that incorporate data, tools, and functions geared toward individual health. In another definition proposed by the Markle Foundation [33], PHR is an e-application enabling individuals to access, manage, and share their health information and that of others for which they are authorised electronically, securely, privately, and confidentially.

Moreover, an individual or authorised person develops, owns, updates, and manages personal health records [34]. Personal health records summarise an individual’s lifelong health history based on procedures, major illnesses, allergies, blood pressure, data collected from home monitoring devices, family history, immunisations, medications, laboratory test results, and information about an individual’s health history [35,36,37]. Health records access can be used through tools and functionalities to manage one’s health and enable communication and record sharing with clinicians [1,8,11,38,39].

This paper defines PHR following Assadi and Hassanein’s [34] definition. Throughout the study, terminologies such as “consumer”, “individual”, and “patient” are interchangeable, as consumers of the PHR system do not necessarily deal with medical information and can be healthy or suffering from illness. PHR system implementation and usage success help facilitate transformative development in the delivery and management of healthcare [37,38,39]. Consequently, patient and care-provider circles are assisted in their effective interaction with further innovation opportunities for care management since patients and care providers share both controls, leading to enhanced and more efficient healthcare.

Regardless of the benefits that consumers can reap from PHR system usage [36,40,41,42,43,44,45], studies have revealed that their adoption has yet to be extensive [39,46,47]. This is attributed by Roehrs et al. [41] to the following challenges that users face: first, there are challenges and issues regarding collaboration and communication that include the storage and availability of data in a PHR as well as limitations regarding the types of information that may be provided by the PHR. In addition, the PHR should be customizable, usable, familiar, and comfortable for the user. The second issue relates to privacy, security, and reliability, including confidentiality, integrity, data repositories and their owners, accessing control protocols, and data transport protocols. A third challenge concerns the infrastructure of PHRs, such as the portability of devices and equipment associated with them. To support PHR solutions, efficient computer systems as well as a scalable infrastructure are necessary. Fourth, there are concerns regarding integration, such as patterns in collecting medical information and terminology used in collecting and storing personal health information. Interoperability is also critical.

To overcome these challenges and barriers, it is necessary to link technology with human behaviour issues in order to gain a better understanding of the adoption of personal health records by end-users, which will ultimately lead to higher adoption rates [48]. Considering the potential system advantages for end-users and how interested they may be in such a system [34,37,38,39], adoption is a must for the realisation of such advantages akin to any IS [8,16,49], and thus, more studies need to be conducted to shed light on how to increase PHR system adoption rates.

Concerning the above, individuals should have access to their health information and be able to control it through the use of personal health records (PHR) so that they can actively participate in the management of their healthcare and eliminate the role of the passive patient [36,38,40,50,51]. However, users must continuously invest effort into keeping up-to-date accounts to ensure that the system can effectively support them. This effort will reduce the likelihood of inaccurate, incomplete, and outdated records in the system, as these may lead to erroneous decisions [31].

An example of an emerging class of information system is the patient health record system, which offers access to and monitoring of useful information that is accompanied by the requirement for ongoing maintenance (for example, regular updates of a patient’s health records), thereby supporting an individual’s active role within the context for which the information system was designed [32,36,38,43,44,45]. Healthcare system users should have the ability to be more proactive in managing the information systems, must reflect suitable personal traits, and take support from the factors in the environment to promote their active role [8]. This facilitates a sufficient motivation level towards system use regardless of continuous maintenance [52].

However, despite industry predictions about increasing consumer interest and government commitment to PHR technologies, their adoption has yet to peak and continues to fall short of expectations. The expression “PHR paradox” has been used to explain the disconnection between active interest and low usage rates of PHRs [53]. Several reasons have been proposed for the lag in adoption in the literature, which are often contradictory with intuition; often, the results are mixed [38,54,55]. As a result, authors have urged more studies in the consumer adoption PHR area [34,37,38,56,57].

More specifically, little information about health technologies in Saudi Arabia is available due to the lack of research [58]. Several studies have overlooked the perspective of healthcare consumers (users) concerning implementing and using an integrated PHR system at the national level [39]. Therefore, this study extends TAM by examining the factors influencing healthcare consumers’ usage of personal health records.

### 1.1. Literature Review

#### PHR Adoption

The literature on PHR has indicated that adoption barriers may be linked to technology factors such as security concerns, system usability, and ineffective healthcare provider system integration [37,41,59,60,61]. Several personal factors have also been articulated as barriers to adopting these technologies, such as lack of technology awareness, competency, chronic medical conditions, and unrealistic expectations [41,57,61,62,63,64,65]. Although several of these factors have been empirically validated, there is often a lack of consistency in the results between studies [41,55,61,66,67,68,69]. The reviewed relevant studies show that chronically ill or disabled patients and their caregivers and older people’s caregivers have a higher likelihood of adopting and using PHR technologies [70,71,72,73]. This user group often views PHR technologies as useful in communicating with the correct personnel to obtain personalised care [37,71,74].

According to a recent study, several factors contribute to PHR adoption, namely computer anxiety, concerns about privacy and security, and perceptions of usefulness, among others [54,55,68,75,76,77,78]. Meanwhile, studies focusing on several adoption factors, including health literacy among consumers, user self-efficacy, and usability perceptions, have shown mixed or contradictory results when evaluating adoption [54,55,79]. Regarding major areas that need more investigation, the review showed that PHR adoption has yet to be thoroughly examined.

In this regards, the study aim was to explain factors such as privacy, security, and usability (exogenous predictors of TAM) to offer insight into the utilization and adoption of and personal health records. The current study contributes significantly to the technology-acceptance literature in two distinct manners. Firstly, is that it is the first to examine the use of PHR in Saudi Arabia, including the extension of the TAM model, and second, it creates a context-driven model that focuses on the associations among privacy, security, and usability and personal health records utilization. Additionally, this study fills a gap in the literature regarding the moderating effects of privacy influence on the relationship between perceived ease of use and intention to use. Further, the moderating effects of usability on the relationship between perceived ease of use, perceived usefulness, and intention to use were investigated. The proposed model enriches information and knowledge regarding the acceptance of PHRs in developing nations, and by doing so, it helps satisfy calls for contextual theorising in the information systems field. The next section of the article presents a description of the proposed model and the constructs and hypotheses that are relevant to it.

### 1.2. Theoretical Foundation

This study adopted TAM as the underpinning theory owing to its influential and effective nature in shedding light on technology usage behaviour [16,18,80,81,82,83,84,85,86]. TAM posits that technology use behaviour, referred to as the behaviour inclination towards accepting technology, can be measured through a user’s attitude towards using technology [87]. Two main attitude predictors towards usage have been proposed: perceived usefulness and easiness [87,88]. The first refers to the belief of an individual that using technology can promote performance of task; the second defines the perception of an individual that technology use is free from effort [87,88]. Additionally, perceived easiness indirectly influences perceived usefulness attitudes [16,18]. Studies have found that TAM can effectively explain differences in technology use behaviour in different contexts and situations, including the health context, for eHealth records (EHRs) [89], telehealth [90], mobile health technologies [16,18], cloud-based services [91], medical devices and telemonitoring tools [92,93], and assistive technology [94].

However, despite the comprehensive inspection and validation of models in terms of health information systems among health professional staff, such examinations do not address consumers’ acceptance of health information systems [16,18,95,96,97], and based on the provided evidence, such acceptance may vary from that of professionals with self-efficacy and experience, as a result of which challenges may be faced during system use [12,16,95,98]. Hence, searching for ways to enhance PHR acceptance among consumers is pertinent.

Additionally, TAM has the same weaknesses as other technology acceptance models, the first of which is that it depends on other factors to determine the attitude of individuals. In general, TAM has been widely employed to investigate internal motivations rather than external ones, as its focus is on the outcomes of IT use. The use process has been largely overlooked, highlighting the need to include external factors in the model. Consequently, a TAM extension with new variables may be able to explain PHR adoption. This study included privacy, security, and usability to extend TAM.

Both privacy and security have been researched in literature, with increasing evidence validating their influence [99,100,101,102,103,104,105]. Based on a systematic review of PHR privacy policies, users are not provided with detailed descriptions of the security issues and adherence to standards and regulations when it comes to a PHR system [104,106]. This may be exemplified by the significant advantages of PHR use and systems privacy risks, with emphasis confined to general privacy and trust issues [99,101,104]. Both security and privacy are major challenges in protecting health information systems, and even though the system’s success depends on various factors such as organisational, technical, and political issues, the authentication and cryptographic management (privacy and trust issues) for prevention of hacker attacks and unauthorised use is of major importance [99,104].

Added to the above, results show that new system usability and its design and user experience contributes to influence system acceptance, and in this regard, the usability of a system can be defined as the amount of effort that must be expended to use it. In general, usability is the degree to which users can effectively and efficiently use a product and the extent to which they are satisfied that they will achieve specific goals through employing a product. It is key to the use of acceptance of PHR, as evidenced by prior studies [32,41,66], to facilitate PHR’s ease of use through the user interface and patient support [38,107,108]. Thus, PHR stakeholders, including designers and developers, should focus on usability aspects.

### 1.3. Proposed Model and Hypotheses Formulation

#### 1.3.1. Perceived Usefulness (PU)

The degree to which an individual believes that a particular technology will improve his or her job performance to an extent that includes enhancing efficiency and effectiveness [88] can be determined by the perceived usefulness of the technology. Based on TAM studies [88,109], perceived usefulness is one of the top technology adoption determinants [110,111]. It is therefore expected that the perceived usefulness of PHR systems will serve a key role in deciding whether they are adopted. Past studies of this calibre have confirmed the key role of perceived usefulness in adoption prediction [55,68,79,112]. In this regard, the first hypothesis is reported:

**Hypothesis** **1 (H1).** *The intent to use PHR is positively influenced by PU*.

#### 1.3.2. Perceived Ease of Use (PEOU)

The level to which an individual believes that using a specific technology will be effort-free is known as PEOU [88]. In this study, PEOU is described as a user’s belief that PHR use is free from mental and physical effort. Studies in the literature dedicated to the PEOU–intention to use PHR relationship generally confirmed the relationship [113,114]. Further, PEOU’s significant influence over PU and intention towards using PHR [27,113] was reported. In this regard, the second and third hypotheses are reported:

**Hypothesis** **2 (H2).** *The PU of PHR is positively affected by PEOU*.

**Hypothesis** **3 (H3).** *The intent to utilize PHR is positively affected by PEOU*.

#### 1.3.3. Intention to Use

New technology acceptance is primarily set by intention towards using such technology, defined as an individual’s desire to engage in a particular behaviour [115]. When referring to the use of PHRs, the intention is a plan towards using it, and according to Hsieh et al. [114], intention towards PHR usage significantly relates to its actual use. In this regards, the fourth hypothesis is reported:

**Hypothesis** **4 (H4).** *PHR usage is positively influenced by the intention to use it*.

#### 1.3.4. Privacy and Security

An essential research topic relative to technology acceptance is the role of privacy and security and the related empirical findings [99,100,101,102]. More specifically, information privacy is the ability of an individual to manage their personal information in light of interactions and exchanges with others [116,117]. Healthcare providers generally manage users’ personal data and provide it to other personnel; owing to this sharing, there is the utmost concern for privacy [118]. Currently, using electronic communication has become common, adding to the privacy, confidentiality, standardisation, and accuracy of PHR [119,120]. A related study by Kaelber et al. [121] indicated that the top concern among patients regarding electronic healthcare applications of every type is security and privacy, which holds true for PHR. In another study, Featherman and Fuller [122] stated that privacy concerns are the focus of potential e-services adopters.

Moreover, based on a systematic review of PHR privacy policies, most such policies failed to provide users with detailed descriptions of security issues and adherence to standards and regulations [106]. In the case of perceived benefits that can be reaped from PHR, the highlights are placed on privacy and trust issues rather than the potential system-related privacy risks [101]. It was found that 67% of people were concerned with their personal medical records privacy (Bishop et al. [123]), indicating the importance of privacy from the patient’s viewpoint [124]. Privacy negatively influences adopting an eHealthcare system, according to Angst and Agarwal [118], while Li et al. [101] revealed that privacy could not completely explain the intention to adopt. Nevertheless, other studies such as that by Whetstone and Goldsmith [125] found that healthcare innovativeness, privacy concerns, and perceived usefulness were the top predictors of adoption intent.

According to Sabnis and Charles [126], security is a determining factor in the decision to adopt web-based PHR, along with confidentiality and privacy. If people are convinced that their personal information is shared privately and is stored in a way that unauthorised parties will not be privy to it [127], their concerns will be assuaged. However, the more individuals who adopt web-based PHR, the higher the risk of breach the information; therefore, privacy and security are main concerns for protecting health systems. Successful systems depend on various factors (organisational, technical, and even political), but authentication and cryptographic management are of top importance for preventing unauthorised use and attacks made by hackers [99]. In this regards, the fifth hypothesis is reported:

**Hypothesis** **5 (H5).** *Security has a positive influence on the intention to use a PHR*.

In connection to the above, patients will be more inclined to use PHR due to its ease of use and the PHR providers’ assurance that the system is credible and capable of minimising privacy risk, which would lead to higher intention towards PHR usage. Hence, the sixth hypothesis is reported:

**Hypothesis** **6 (H6).** *Privacy moderates the relationship between PEOU and the intention to use a PHR*.

#### 1.3.5. Usability

The usability concept may be defined as the effort needed towards using a computer system. According to Nielsen [128], usability is associated with the ease with which a user can learn to manage a system, the ease of learning the fundamental system functions, the level of efficiency with which the site has been developed, the level of error avoidance, and the general user satisfaction when it comes to system management. On the whole, usability reflects how users can use a particular system [105], and thereby, high system usability is related to lower difficulty levels of managing its functions [88]. Usability has always been considered a major predictor of intentions towards system usage [129].

The following statements can summarise website usability:

- Easy understanding of the system structure, functions, interface, and content by a user;
- Simple use of the initial stages of a website,
- Speedy search for required information,
- Ease of browsing in light of the time and work required to obtain the expected results,
- User’s ability to control and navigate the system at any time.

Regarding health information systems, usability issues have garnered significance in system rejection/acceptance, as evidenced in computerised patient records that depend on the system’s usability [130,131,132,133,134]. Evidence points to the fact that issues surrounding usability directly influence patient outcomes, including opportunity cost, while other issues that indirectly impact usability include coping strategies in dealing with software problems and limitations and complexity and that entail dealing with complexity strategies, breach of communication and usability of software, oversight of bias, and usability on patient safety [131,132,133,134,135]. In this day and age, consumers are faced with an extensive array of personal health information-management tools at their convenience, and PHR’s ability to satisfy their needs depends at some level on the way product designers focus on users’ needs and a user’s involvement in the design, testing, and system re-design.

Usability of PHR indicates the perceived ease of managing a site or accessing and keeping track of health information online, and this is deemed a major factor in PHR development. Patients’ willingness to accept depends on the user-friendliness of the PHR system and ease of learning usage and browsing. Meanwhile, a complicated system could only lead to human error and dissatisfaction among the users, and eventually, rejection rather than acceptance will be the outcome [136]. Additionally, patients will be convinced that PHR usage is easy when they can easily learn system management and memorise fundamental system functions. This would lead to higher intention towards PHR usage; in other words, PEOU and PHR intention towards use will be correlated more strongly with higher PHR usability, and in this regard, the seventh hypothesis is reported:

**Hypothesis** **7 (H7).** *The relationship between PEOU and intention to use personal health records is moderated by usability*.

Additionally, if patients are convinced that PHR use will enhance their health status and quality of health services through its efficient functions and design, they will readily accept it, with higher intention towards its usage. The higher the usability of PHR, the higher the relationship between PU and intention towards PHR usage. In this regards, the eighth hypothesis is reported:

**Hypothesis** **8 (H8).** *The relationship between PU and intention to use personal health records is moderated by usability*.

**Figure 1:**
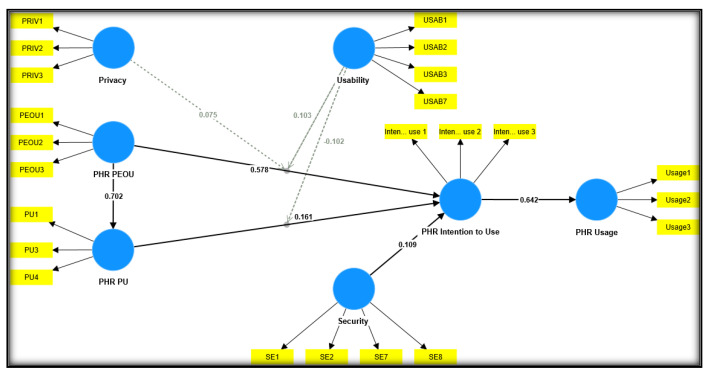
Research Model.

Figure 1 offers a theoretical framework overview explaining how TAM, privacy, security, usability, and PHR use are related in terms of each other.

## 2. Materials and Methods

### 2.1. Research Context

The launching of the Kingdom of Saudi Arabia’s national eHealth strategy in 2011 is in line with Vision 2030, which is a roadmap for the country’s economic growth and development [137]. The strategy covers the National Transformation Program, among which are eight themes of enhancing healthcare services quality and efficiency through a patient-centred healthcare culture and enhanced patient involvement using technology [138]. With the introduction of eHealth to the Kingdom of Saudi Arabia healthcare, researchers have initiated research into its different aspects [39,51,139,140,141,142,143,144,145,146,147]. Most studies have examined the influencing factors on intention towards PHR use at the pre-adoption stage, while a few focused on the influencing factors at the usage stage. For instance, Al-Sahan [143] examined the perceived hindrance or challenges towards PHR adoption in the Ministry of National Guard Health Affairs (MNGHA) based on two perspectives: technical and social. Based on the results garnered using 424 patients, a positive perception of PHR adoption existed, constituting 96.7%, which shows the avid interest of the patients in PHR usage, and the majority of them, constituting 73.3%, expressed no concerns for confidentiality when accessing their healthcare information online.

Similarly, Saudi Arabian patients’ perspectives and expectations were tackled by Alhur [51] in his study of PHR, which found participants to be highly interested in the system compared to other studies in developed nations. Most were inclined towards PHR use, perceiving them as valuable to health, albeit some expressed security concerns regarding online records. Overall, the patients were generally optimistic in their view of PHR in enhancing their privacy online. The two studies were descriptive without the employment of TAM, UTAUT, or other IS theories.

Hence, in the present study, the objective was to examine privacy, security, and usability (exogenous predictors of TAM) and their influence on PHR use. The research seeks to contribute to the literature concerning technology acceptance in two major ways: the first of which is to be among the few studies that used TAM to examine PHR use in Saudi Arabia, and the second of which is developing a context-driven model to investigate the relationship between privacy, security, and usability and using PHR. The hope is that the proposed model provides insight into the PHR acceptance domain in the context of a developing nation, responding to the need to carry out contextual theorisation in IS studies.

### 2.2. Sample and Data Collection

This quantitative study uses a cross-sectional design to examine the proposed model. The study used a questionnaire survey as the main data collection instrument, and copies were distributed to King Abdul-Aziz University faculty members, employees, and students that own a personal healthcare record (Shifaa platform). Surveys were developed in English and then translated into Arabic, as Arabic is the mother tongue of the Saudi people. After translation, an online-based questionnaire was distributed through a survey link to selected respondents via a university email distribution group. A social media platforms were used to share the survey link with the involvement of university communities. The data collection lasted 2 months, from 30 June to 30 August 2022. Following Krejcie and Morgan’s [148] table, it was determined that 384 respondents would be an appropriate size. The study retrieved 389 survey results; upon scrutiny, they were all found to be complete and useable for analysis.

As mentioned, the English version of the original survey was translated into Arabic—this was necessary, as adopting validated instruments from past studies saves time and effort compared to developing a survey from scratch. A 5-point Likert scale was employed to measure survey items, and the scales were adopted from past literature (see Appendix A for measurement items).

The demographic information analysis showed that the respondents were mostly male, totalling 221 of the population (56.9%), and the majority of their ages ranged from the 17- to 25-years category (216 respondents), constituting 55.5% of the total respondents with bachelor degrees (49.1%). Moreover, 257 respondents, constituting 66% of the total respondents, had experience using the system for less than a year. Table 1 tabulates the demographic characteristics analysis results, covering the respondents’ age, education level, gender, and experience using the system.

## 3. Results

This study employed partial least squares (PLS)–SEM to test the proposed framework, enabling the measurement and structural models to be examined simultaneously [149,150,151]. As well as being effective in addressing complex models with hierarchical structures, PLS is also highly effective in dealing with models with multiple relationships, indicators, and constructs [152,153,154,155,156,157]. In addition, PLS can be used to deal with problems that may arise because of small sample sizes and errors, as it only relies on a few rigid assumptions regarding the normal distribution of data to deal with such problems [84,152,158,159,160]. PLS Version 4.0.8.4 was employed to test the proposed model, with the first step involving testing the measurement model’s reliability and validity [159,161,162]. AVE, indicator reliability, internal consistency, and discriminant validity, which other authors previously proposed, were employed to determine whether the study had convergent validity [8,16,84,163,164,165,166]. Table 2 contains the composite reliability (CR) values, item loadings, Cronbach’s alpha (CA), and constructs AVE. The table shows that all CA values exceeded 0.60, which were all acceptable based on Pallant [167] and Nunnally and Bernstein [168], and CR values exceeded 0.70 throughout the constructs, which confirmed internal consistency and appropriate nature of constructs based on Hair et al. [166,169,170]. According to the results, the items of the constructs had a reliability of greater than 0.40, which was sufficient for them to be considered acceptable [170].

In terms of convergent validity, all AVE values exceeded 0.50, which was the threshold value [170]. Based on the squared AVE values of the constructs, the conclusion can be reached that the discriminant validity of the constructs exceeded the threshold value. Based on the fact that all the values were higher than the correlation construct values, the discriminant validity of the constructs was confirmed by the fact that all the values were higher than the correlation construct values [170] (See Table 3).

### Perceived Usefulness (PU)

The structural model and hypotheses analysis was conducted using a main effect model, whereas an analysis of the moderation effect was carried out using an interaction model [163,170]. To generate the path coefficient and evaluate the significance of the effects of the study models, the PLS path algorithm was applied to the study models’ outputs. Based on past studies’ recommendations [159,169,170], 5000 bootstrapping resamples were applied (refer to Figure 1), and the path coefficients significance was determined for direct effects (refer to Table 4) and moderating effects (refer to Table 5).

Consistent with the study’s hypotheses, the results in the above table show the significant and positive impact of perceived usefulness on intention to use PHR (β = 0.161, t = 2.595, *p* < 0.005), supporting H1. As for perceived ease of use, a significant and positive effect was found on perceived usefulness to use PHR (β = 0.702, t = 21.406, *p* < 0.00), supporting H2, and perceived ease of use was also found to significantly influence PHR intention to use (β = 0.578, t = 10.527, *p* < 0.00), and thus, H3 was also supported. Intention to use PHR positively and significantly influenced PHR actual use (β = 0.642, t = 18.831, *p* < 0.00), confirming and supporting H4. Lastly, security positively and significantly influenced the intention to use a PHR (β = 0.109, t = 2.877), thus supporting H5.

**Figure 2:**
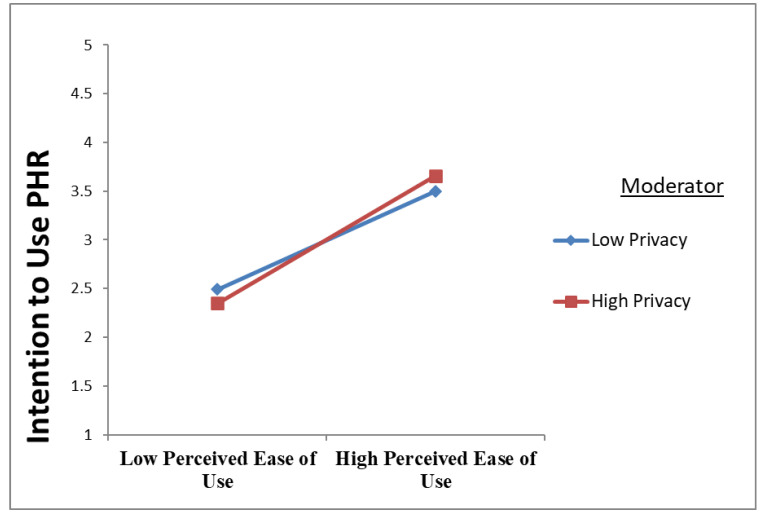
Privacy’s Positive Moderating Role on the Association between Perceived Ease of Use and Intention to Use PHR.

The moderating hypotheses (H6, H7, and H8) concerning privacy and usability were examined using an interaction model. The study created three latent interaction constructs to depict the interaction between PU and PEAU (TAM-related factors and the moderating constructs (privacy and usability) and their influence on PHR intention to use, i.e., the criterion variable. A bootstrapping procedure with 5000 resampling was used, and Table 5 contains the detailed results. Based on the positive moderation path coefficient of the interaction term between privacy and perceived ease of use (β = 0.075, t = 2.008, *p* < 0.020), privacy positively moderated the PEOU–intention to use the PHR relationship, supporting H6. In essence, privacy moderated the PEOU–PHR intention to use relationship. As shown in Figure 2 and Table 6, privacy is a moderator in maintaining the association between perceived ease of use and intention to use PHR. Privacy strengthens the positive association between perceived ease of use and intention to use PHR.

**Figure 3:**
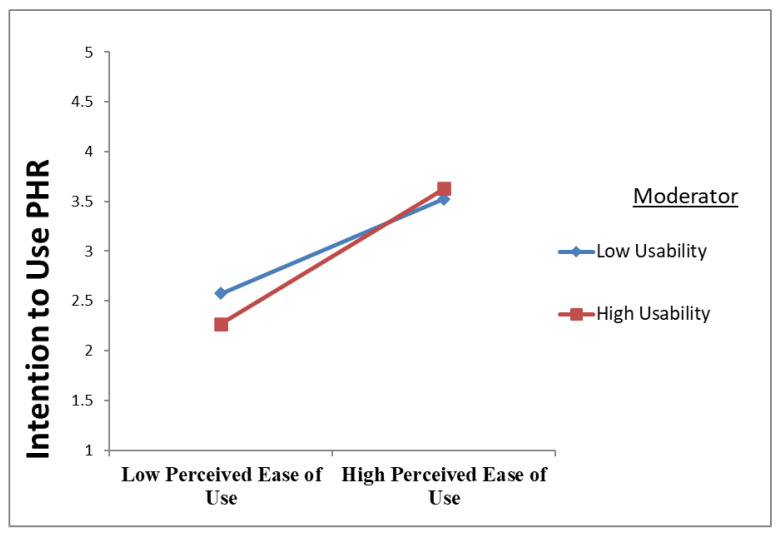
Usability Positively Moderated the Relationship between Perceived Ease of Use and Intention to Use PHR.

Moving on to the moderating effect of usability, the moderation path coefficient result (β = 0.095, t = 1.903, *p* < 0.029) shows that usability moderates the association between perceived ease of use and PHR intention to use, confirming H7. The result is presented in Figure 3 and Table 7. The moderating effect supports the positive association between perceived ease of use and PHR intention to use.

**Figure 4:**
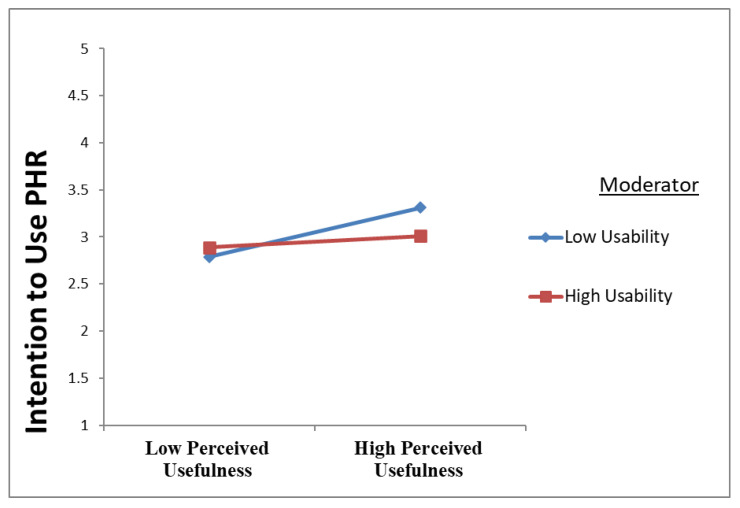
Usability Lessens the Positive Relationship between Perceived Usefulness and Intention to Use PHR.

The negative moderation path coefficient of the interaction term between usability and perceived usefulness (β = −0.102, t = 1.747, *p* < 0.04) is indicative of the negative moderating effect of usability on the association between perceived usefulness and PHR intention to use, and as such, H8 is also supported. The results are demonstrated in Figure 4 and Table 8. The results show that the usability of PHRs reduces the positive association between perceived usefulness and PHR intention to use.

Finally, the PLS structure model was evaluated using the criterion coefficient of determination (R2). According to Sarstedt et al. [159], the rule of thumb when it comes to R2 values is such that 0.67 is deemed substantial, 0.33 is moderate, and 0.19 is weak. These findings revealed that TAM integration was successful in predicting PHR use, and with such addition, the model’s predictive power increased and managed to explain 0.492 of the variances in patients’ PHR usage and 0.548 of the variance of intention towards use. The study model succeeded in explaining 0.492 of the perceived usefulness perception of patients towards PHR.

## 4. Discussion

This research validates the accuracy of TAM in predicting the use of PHR among patients by supporting its assumptions with additional variables, thus strengthening the model’s predictive abilities.

The results found supported the significant association between perceived usefulness and consumers’ intention to use PHR (*p* < 0.05), which is in line with past reported studies on eHealth adoption, including Alsyouf et al. [16,18], as well as PHR adoption, including Noblin et al. [171], Abdekhoda et al. [103], and Liu [27]. These studies supported PU’s role in driving users’ behavioural intention towards PHR use. In other words, if patients believe that PHR can provide benefits, they will use it to enhance healthcare services. Healthcare quality is enhanced through this technology by eliminating waiting times, and through health profile management, users can also maintain a higher rate of health profile usage and management.

Added to the above, a significant association was found between perceived ease of use and perceived usefulness (*p* < 0.00), as with the other prior literature on eHealth adoption such as that of Alsyouf et al. [16,18] and on PHR adoption such as that of Abdekhoda et al. [103], Liu [27], and Noblin et al. [171]. Stated clearly, people who find PHRs easy to use are more likely to use them frequently, supporting their perception of their value and importance.

Moreover, the study findings supported a significant association between PEOU and PHR intention to use (*p* < 0.00), as proposed in H3 and as revealed by past literature, including Alsyouf et al. [16,18] in the eHealth context and Abdekhoda et al. [103] and Elsafty et al. [172] in PHR adoption context. Based on this result, perceived ease of use among patients concerning PHR could result in increased intention towards using the system and ultimately their actual use. This result can be attributed to the importance of PEOU in PHR among patients. According to the literature, consumers’ acceptance of health informatics applications differs from that of health professionals [16,95,98]. As a result of the challenges they have experienced in using the system, consumers have a low level of self-efficacy and a negative perception of the system’s usability. Therefore, it is necessary to assist patients in accepting PHRs.

Moving on to the fourth hypothesis, which proposed PHR intention influence over actual PHR use, a positive influence was found (*p* < 0.00), which is in line with past studies’ findings [8,11,103]. In other words, users’ behavioural intention indicates their acceptance and actual use of technology—their intention towards PHR use is a predictor of their actual use, similar to the finding in the eHealth context reported by past literature.

Security was found to have a significant association with PHR intention to use (*p* < 0.04), supporting H5, and this significant relationship was also found by Saigi-Rubio et al. [173]. If people are convinced that their personal information is shared safely, far from manipulation by unauthorised individuals [127], their adoption of PHR will increase.

In the sixth hypothesis (H6), privacy was proposed to moderate the association between perceived ease of use and intention to use PHR, and the hypothesis was supported, indicating that privacy heightens the influence of perceived ease of use on PHR intention to use. This may be attributed to the patient’s belief that using PHRs will become easier when providers of the system can minimise privacy risks and their effects, thus contributing to higher intention to use the system.

Hypothesis 7 proposed the moderating effect of usability on the perceived ease of use–intention to use PHR relationship, and the findings supported it. Privacy with PEOU determines the level of PHR use among patients in that patients who are convinced that PHR use is easier when they can learn system management and memorise the basic functions would intend to use it. The higher the PHR usability, the stronger the PEOU–intention to use PHR relationship.

Finally, in the eighth hypothesis (H8), usability was posited to moderate the PU–intention to use the PHR relationship. Usability was found to influence the relationship between the two negatively. This result may be attributed to the notion that if patients believe that the PHR system falls short of meeting their needs owing to deactivated services or improper working of services, this would be perceived as weak system functionality; eventually, usability would harm the PU–intention to use PHR relationship.

## 5. Conclusions

In literature, TAM has often been used and adopted to examine various eHealth application types in different contexts. In this study, the focus is placed on PHR use, assuming that if users have a positive intention towards PHR usage in light of its usefulness, ease of use, usability, privacy, and security, they will increase their use and acceptance of it. TAM was adopted as the underpinning model to examine the study variables and to understand why eHealth applications in general, with PHR in particular, have not been extensively adopted in Saudi Arabia. Three exogenous variables, namely privacy, security, and usability, were added to TAM to examine existing values, past experiences, and needs of potential users. SEM analysis showed that the model could explain the predictive ability of the variables of PHR intention to use and actual use. Perceived usefulness, perceived ease of use, and security were found to be relevant in their direct influence on intention towards PHR use, while privacy is relevant in terms of its moderating effect on the PHR PEOU and PHR intention to use relationship. Usability was also relevant in positively moderating the PHR PEOU and PHR intention to use relationship. However, usability had a negative moderating effect on the PHR PU–PHR intention to use relationship.

### Limitations and Future Research

This study has several limitations. The first limitation is the nature of the study. A cross-sectional survey requires accounting for the differences among the relationships across divisions, locations, contexts, and countries, as the meaning may disappear over time. In this case, future studies may adopt a longitudinal study instead. The second limitation is the data collected through email distribution to university members, specifically to one of the biggest Saudi universities—but a single university nonetheless, which limits the outcome’s generalizability. While the study’s target population (students, employees, and faculty members) limited the generalizability of the findings, it does provide insight into how PHR are used by a very large segment of society, which drives IT adoption in a community. Furthermore, PHRs are not only used by people who are ill but also by healthy individuals. This study is expected to pave the way for future studies that will include other segments of society. By doing so, we will be able to gain a deeper understanding of the adoption of information systems, specifically PHRs. To this end, future studies may take different settings and employ large-sized samples representing the same context.

Moreover, future studies may adopt data collection methods other than the survey questionnaire to enable comparative studies or assessments of pre-adoption and post-adoption behaviours that are valuable for health applications. A qualitative approach would also enable the acquisition and observation of life experiences, which are crucial to positional analysis—this is possible through the elicitation of narrative analysis or the explanation of the phenomenon. Future studies may also extend TAM through other external variables not examined in the study, such as the self-efficacy of technology, quality factors (the quality of service, the quality of the system, and the quality of the information), as well as satisfaction with the technologies, which are all important factors. Additionally, age, gender, and other demographic characteristics may be addressed. Notably, this study adopted TAM solely without its integration with other theories and models—future studies may integrate them and re-examine the study findings to enrich the literature and, ultimately, practice. Accordingly, this study recommends that the Population-Intervention-Environment-Transfer Model of Transferability (PIET-T) be integrated with the TAM in order to develop a wider understanding of user acceptance of official systems as well as other key elements of the transferability concept.

## Figures and Tables

**Table 1:** Demographic characteristics of the respondents.

Demographic Characteristics	Category	*n*	%
Gender	Male	221	56.9
	Female	168	43.1
	Total	389	100
Age	17–25 years old	216	55.5
	26–41 years old	58	14.9
	42–57 years old	96	24.7
	58–67 years old	19	4.9
	Total	389	100
Education level	High school degree and below	78	20
	Bachelor’s degree	191	49.1
	Master’s degree	22	5.8
	Ph.D. holders	98	25.1
	Total	389	100
System usage experience	Less than one year	257	66
	From 1–3 years	83	21.3
	From 4–7 years	49	12.6
	Total	389	100

**Table 2:** Item loadings, Cronbach’s alpha values, composite reliability values, and AVE values.

Construct	Measurement Items	Loadings	Cronbach’s Alpha	Composite Reliability	Average Variance Extracted (AVE)
PHR IU	IU 1	0.958	0.935	0.935	0.884
	IU 2	0.920			
	IU 3	0.943			
PHR PEOU	PEOU1	0.882	0.866	0.869	0.788
	PEOU2	0.908			
	PEOU3	0.874			
Privacy	PRIV1	0.927	0.906	0.917	0.841
	PRIV2	0.934			
	PRIV3	0.890			
PHR PU	PU1	0.919	0.918	0.919	0.860
	PU3	0.936			
	PU4	0.927			
Security	SE1	0.764	0.69	0.738	0.512
	SE2	0.671			
	SE7	0.823			
	SE8	0.58			
Usability	USAB1	0.899	0.934	0.948	0.835
	USAB2	0.908			
	USAB3	0.911			
	USAB7	0.937			
PHR Usage	Usage1	0.878	0.905	0.909	0.841
	Usage2	0.953			
	Usage3	0.919			

**Table 3:** Construct Discriminant Validity.

	PHR IU	PHR PEOU	PHR PU	PHR Usage	Privacy	Security
PHR IU	0.940					
PHR PEOU	0.711	0.888				
PHR PU	0.581	0.702	0.927			
PHR Usage	0.642	0.516	0.403	0.917		
Privacy	−0.100	−0.095	−0.096	−0.018	0.917	
Security	0.214	0.141	0.164	0.231	−0.097	0.716
Usability	−0.077	−0.014	−0.049	−0.017	0.289	−0.063

**Table 4:** Direct Relationship Hypotheses.

No	Hypothesis	Beta	*p*-Value	t-Statistic	Decision
H1	Perceived Usefulness→Intention to Use PHR	0.161	0.005	2.595	Accepted
H2	Perceived Ease of Use→Perceived Usefulness	0.702	0.00	21.406	Accepted
H3	Perceived Ease of Use→Intention to Use PHR	0.578	0.00	10.527	Accepted
H4	Intention to Use PHR→PHR Actual Use	0.642	0.00	18.831	Accepted
H5	Security→Intention to Use PHR	0.109	0.002	2.877	Accepted

**Table 5:** Moderating Effects.

No	Hypotheses	Beta	Standard Error	t Value	Decision
H6	Privacy × PHR PEOU→PHR Intention to Use	0.075	0.037	2.008	Accepted
H7	Usability × PHRPEOU→PHR Intention to Use	0.103	0.054	1.903	Accepted
H8	Usability × PHR PU→PHR Intention to Use	−0.102	0.058	1.747	Accepted

**Table 6:** Two-way Interaction Effects for Unstandardised Variables (PEOU, Privacy, and Intention to Use PHR).

Variable Names:	
Independent Variable:	Perceived Ease of Use
Moderator:	Privacy
Dependent variable:	Intention to Use PHR
Unstandardised Regression Coefficients:	
Independent variable:	0.578
Moderator:	0.004
Interaction:	0.075

**Table 7:** Two-way Interaction Effects for Unstandardised Variables (PEOU, Usability, and Intention to Use PHR).

Variable Names	
Independent variable:	Perceived Ease of Use
Moderator:	Usability
Dependent variable:	Intention to Use PHR
Unstandardised Regression Coefficients:	
Independent variable:	0.578
Moderator:	−0.050
Interaction:	0.103

**Table 8:** Two-way Interaction Effects for Unstandardised Variables (PU, Usability, and Intention to Use PHR).

Variable Names	
Independent variable:	Perceived Usefulness
Moderator:	Usability
Dependent variable:	Intention to Use PHR
Unstandardised Regression Coefficients:	
Independent variable:	0.161
Moderator:	−0.050
Interaction:	−0.102

## Data Availability

The data presented in this study are available on request from the corresponding author.

## Appendix A

**Table A1:** Variables with measurement items factors.

**Security**	**Items**	**Reference**
SE1	I think PHR system (Shifaa platform) has mechanisms to ensure the safe transmission of its users’ information.	[174,175]
SE2	I think PHR system (Shifaa platform) shows great concern for the security of any transactions.	
SE3	I think PHR system (Shifaa platform) has sufficient technical capacity to ensure that no other organisation will supplant its identity on the Internet.	
SE4	I am sure of the identity of PHR system (Shifaa platform) when I establish contact via the Internet.	
SE5	When I send data to PHR system (Shifaa platform), I am sure that they will not be intercepted by unauthorised third parties.	
SE6	I think PHR system (Shifaa platform) has sufficient technical capacity to ensure that the data I send will not be intercepted by hackers.	
SE7	When I send data to PHR system (Shifaa platform), I am sure they cannot be modified by a third party.	
SE8	I think PHR system (Shifaa platform) has sufficient technical capacity to ensure that the data I send cannot be modified by a third party.	
**Privacy**	**Items**	**Reference**
PRIV1	I think PHR system (Shifaa platform) shows concern for the privacy of its users.	[175,176]
PRIV2	I feel safe when I send personal information to PHR system (Shifaa platform).	
PRIV3	I think PHR system (Shifaa platform) abides by personal data protection laws.	
PRIV4	I think PHR system (Shifaa platform) only collects user personal data that are necessary for its activity.	
PRIV5	I think PHR system (Shifaa platform) respects the user’s rights when obtaining personal information.	
PRIV6	I think that PHR system (Shifaa platform) will not provide my personal information to other companies without my consent.	
**Usability**	**Items**	**Reference**
USAB1	In PHR system (Shifaa platform) everything is easy to understand.	[177,178,179]
USAB2	PHR system (Shifaa platform) is simple to use, even when using it for the first time.	
USAB3	It is easy to find the information I need from PHR system (Shifaa platform).	
USAB4	The structure and contents of PHR system (Shifaa platform) are easy to understand.	
USAB5	It is easy to move within PHR system (Shifaa platform).	
USAB6	The organisation of the contents of PHR system (Shifaa platform) makes it easy for me to know where I am when navigating it.	
USAB7	When I am navigating PHR system (Shifaa platform), I feel that I am in control of what I can do.	
**Usage**	**Items**	**Reference**
Usage1	I have an account on PHR system (Shifaa platform) to manage my health.	[16,18]
Usage2	Currently using the PHR system (Shifaa platform) to manage my health.	
Usage3	Use the PHR system (Shifaa platform) frequently to manage my health.	
**Perceived Usefulness**	**Items**	**References**
PU1	Using the PHR system (Shifaa platform) improves the effectiveness of my health care.	[16,18,27]
PU2	Using the PHR system (Shifaa platform) decreases the complexity of my health care.	
PU3	Using the PHR system (Shifaa platform) increase the quality of my health care.	
PU4	Using the PHR system (Shifaa platform) for my healthcare is a good idea.	
PU5	I find the PHR system (Shifaa platform) useful in my healthcare.	
**Perceived Ease of Use**	**Items**	**References**
PEOU1	I think that the PHR system (Shifaa platform) is flexible to use.	[16,18,27]
PEOU2	Learning to operate the PHR system (Shifaa platform) is easy for me.	
PEOU3	I can easily get the PHR system (Shifaa platform) to do what I want it to do.	
**PHR Intention**	**Items**	**References**
Intention to use 1	I intend to continue using PHR system (Shifaa platform) to manage my health in the future.	[16,18,27]
Intention to use 2	My willingness to use PHR system (Shifaa platform) is high.	
Intention to use 3	I plan to continue to use PHR system (Shifaa platform) in the future.	
